# Despite disorganized synapse structure, Th2 cells maintain directional delivery of CD40L to antigen-presenting B cells

**DOI:** 10.1371/journal.pone.0186573

**Published:** 2017-10-12

**Authors:** Jennifer L. Gardell, David C. Parker

**Affiliations:** Department of Molecular Microbiology and Immunology, Oregon Health & Science University, Portland, Oregon, United States of America; Universite Paris-Sud, FRANCE

## Abstract

Upon recognition of peptide displayed on MHC molecules, Th1 and Th2 cells form distinct immunological synapse structures. Th1 cells have a bull’s eye synapse structure with TCR/ MHC-peptide interactions occurring central to a ring of adhesion molecules, while Th2 cells have a multifocal synapse with small clusters of TCR/MHC interactions throughout the area of T cell/antigen-presenting cell interaction. In this study, we investigated whether this structural difference in the immunological synapse affects delivery of T cell help. The immunological synapse is thought to ensure antigen-specific delivery of cytolytic granules and killing of target cells by NK cells and cytolytic T cells. In helper T cells, it has been proposed that the immunological synapse may direct delivery of other effector molecules including cytokines. CD40 ligand (CD40L) is a membrane-bound cytokine essential for antigen-specific T cell help for B cells in the antibody response. We incubated Th1 and Th2 cells overnight with a mixture of antigen-presenting and bystander B cells, and the delivery of CD40L to B cells and subsequent B cell responses were compared. Despite distinct immunological synapse structures, Th1 and Th2 cell do not differ in their ability to deliver CD40L and T cell help in an antigen-specific fashion, or in their susceptibility to inhibition of help by a blocking anti-CD40L antibody.

## Introduction

B cells act as antigen-specific antigen-presenting cells (APCs) to solicit help from helper T cells (Th cells) in the antibody response [1]. Upon antigen recognition, T cells deliver help in the form of the membrane bound cytokine, CD40L, and other cytokines to the B cells. The CD40L/CD40 interaction is required for the T cell-dependent antibody response. In CD40L- or CD40-deficient mice or after injection of anti-CD40L antibody, antibody formation is suppressed, and germinal centers do not develop [2, 3]. Due to the essential nature of this cytokine in development of adaptive immunity, it is important to determine how this cytokine is delivered in an antigen-specific manner. Targeted delivery of CD40L by helper T cells could limit help to only the antigen-specific, antigen-presenting B cells, and thereby aid in the selection process necessary to develop high-affinity antibodies against foreign pathogens.

T cells release CD40L to the T cell surface with two different kinetics. First, there is a small amount of preformed, intracellular CD40L stored in all Th cell subsets, excluding T regulatory cells, that is mobilized to the cell surface rapidly following brief TCR stimulation [4–6]. Additionally, like other cytokines, CD40L can be produced in large amounts *de novo* from new messenger RNA upon longer interaction with an APC. *In vivo* imaging of germinal centers has proven that most T cell/B cell interactions are brief and not long enough for production of *de novo* protein [7–10]. Therefore, we proposed that TCR-mediated delivery of preformed CD40L allows helper T cells deliver CD40L in brief, antigen-specific interaction *in vivo* [5, 11]. Our recent investigations on the delivery of CD40L have shown that rather than being internalized by T cells following CD40 engagement [12, 13], CD40L is actually transferred in an antigen-specific manner to antigen-presenting B cells [14].

Abraham Kupfer was the first to describe the reorganization of surface molecules at the contact zone between natural killer cells, cytotoxic T lymphocytes, and helper T cells and antigen-presenting target cells [15]. He proposed that this bull’s eye structure, a ring of adhesion molecules surrounding a central zone of MHC and TCR molecules, later termed an immunological synapse, may ensure antigen-specific delivery of effector molecules by these cells.

When naïve Th cells proliferate and generate effector cells, they can be divided into subsets defined by the cytokines they produce. Th1 cells make IFNγ and can acquire cytolytic function, while Th2 cells make IL-4 and IL-5 and are involved in asthma and allergy. We showed that while Th1 cells have the organized bull’s eye synapse structure described by others, Th2 cells have a less well-organized synapse with many foci of TCR/MHC molecules interspersed with regions of adhesion molecules [16]. If the bull’s eye synapse is required for antigen-specific delivery of CD40L to an antigen-presenting B cell, we reasoned that Th2 cells that lack the bull’s eye structure may be unable to deliver CD40L in an antigen-specific manner. In this report, we compare Th1 and Th2 cells for their ability to deliver CD40L to and activate antigen-presenting B cells versus bystander B cells that lack antigen.

## Materials and methods

### Mice

**Fig 1:**
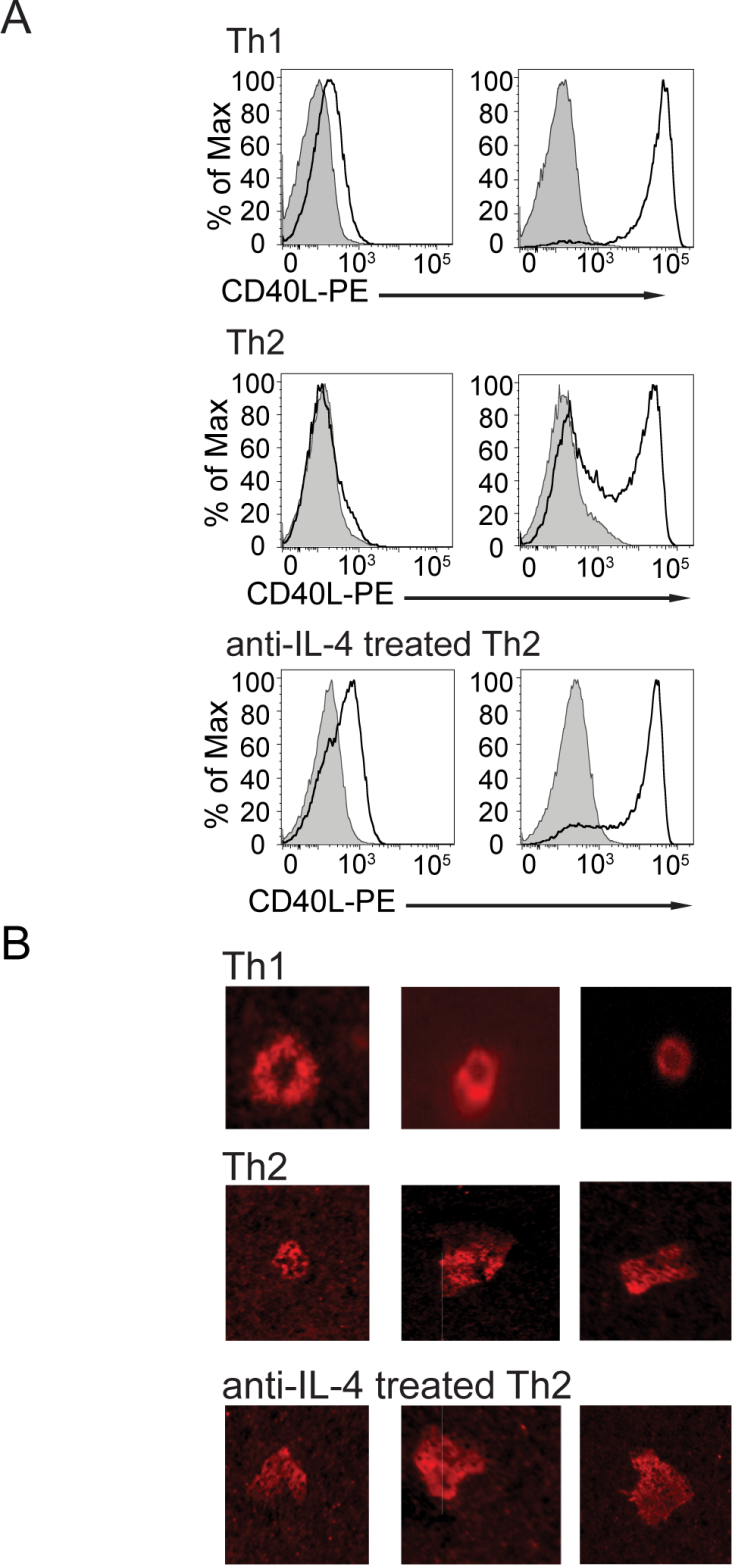
Anti-IL-4 treated Th2 cells retain a pre-formed compartment of CD40L and maintain a multifocal synapse structure. (**A**) CD40L mobilization to the cell surface in 5CC7 Th1, 5CC7 Th2, and anti-IL-4 treated 5CC7 Th2 cells following 30 minutes (left histogram) or 2 hours (right histogram) of PMA and ionomycin stimulation as detected by inclusion of PE-labeled anti-CD40L antibody during stimulation. Control, unstimulated Th2 cells stained with PE-labeled anti-CD40L is shown in gray. (**B**) Three representative 5CC7 Th1, AD10 Th2, and anti-IL-4 treated AD10 Th2 immunological synapse structures are shown. ICAM-Cy5 is shown in red. Unlabeled peptide-bound I-E^k^ was included in the lipid bilayer. Th2 cells and anti-IL-4-treated Th2 cells were scored for formation of a bull’s eye ring of ICAM-1 in three independent experiments: 82 Th2 cells and 50 anti-IL-4-treated Th2 cells were scored in Experiment 1, 650 Th2 and 278 anti-IL-4-treated Th2 in Experiment 2, and 255 Th2 and 237 anti-IL-4 treated Th2 in Experiment 3.

AD10 TCR transgenic mice on a B10.BR background, specific for pigeon cytochrome c 88–104 and reactive against moth cytochrome c 88–103, were generated by S. Hedrick (University of California at San Diego, La Jolla, CA) and acquired from P. Marrack (National Jewish Center, Denver, CO). B10.A (Taconic), B10.A-*Rag2tm1Fwa H2-T18a* Tg (5CC7 TCR transgenic, Taconic), B6.129P2-*Cd40tm1Kik*/J (CD40 knock-out on B6 background, The Jackson Laboratory) and B6.129S2-*Cd40lgtm1Imx*/J (CD40L knock-out on a B6 background, The Jackson Laboratory) were purchased. B10.A CD40 knock-out mice were generated by breeding B10.A mice to CD40KO and selecting homozygous H-2^k^ CD40KO breeders from the F2 generation and subsequent generations. CD40L KO male mice were generated by breeding 5CC7 male mice to CD40LKO females. T cells from AD10 TCR transgenic mice were used to generate the Th2 and anti-IL-4 Th2 immunological synapse structures shown in Fig 1, while all other experimental results were generated using 5CC7 TCR transgenic mice. Mice were housed in specific-pathogen free conditions at Oregon Health and Science University according to institutional standards, and the research protocols, IS00003102 and IP00000656, were approved by the Oregon Health & Science University Institutional Animal Care and Use Committee.

### *In vitro* T cell polarization

Th1 cells were prepared as previously described [14]. To polarize to Th2 cells, spleens were harvested from TCR transgenic mice, and CD4+ T cells were purified using the EasySep system (STEMCELL technologies). CD4+ T cells were cultured in RPMI 1640 medium (Gibco®, Waltham, MA), supplemented as previously described [17], at 0.85 x 10^6^ cells/ml with cesium-irradiated (15 Gray) splenocytes of B10.A mice at 4.15 x 10^6^ cells/ml, MCC peptide at 2.5 μM, IL-4 (50 ng/ml, eBioscience), and anti-IFNγ (20 μg/ml, clone XMG 1.2, BioXcell). IL-2 (derived from the supernatant of a T cell hybridoma cell line) was added to these cells at 80 U/ml two days after culture. Four days after culture, dead cells were removed by centrifuging over Lympholyte M (Cedarlane), and the Th2 cells were restimulated with fresh APCs. During secondary culture, anti-IL-4 was added at 10 μg/ml instead of IL-4 to enable expression of preformed CD40L in the Th2 cells [6].

### *In vitro* overnight assay for T/B collaboration

Th1 and Th2 cells cultures were harvested on day four after stimulation, or restimulation in the case of Th2 cells. Lympholyte M was used to remove dead cells. Cells were then counted and resuspended at 2 x 10^6^ cells/ml either with or without 2 μM Cyclosporin A (CsA) (Sigma-Aldrich) to block *de novo* TCR-induced cytokine production. Spleens were harvested from B10.A and B10.A CD40 knock-out mice, and target splenocytes were prepared by hypotonic lysis. Antigen-bearing splenocytes were labeled with carboxyfluorescein succinimidyl ester (CFSE, Thermo Fisher Scientific) or Cell Trace Violet (CTV, Thermo Fisher Scientific) following manufacturer’s protocol, and pulsed with 2.5 μM MCC peptide for two hours at 37°C. The splenocytes were then were washed three times and combined with 1 x 10^6^ T cells, either with or without 1 μM CsA. Overnight assays were performed in the presence or absence of fluorescently labeled anti-CD40L-PE antibody (clone MR-1, eBiosciences) at 1 μg/ml or unlabeled anti-CD40L (clone MR-1, BioXcel) or Armenian Hamster IgG isotype control (eBiosciences) at the indicated concentrations. After overnight incubation, cells were stained with anti-CD4-PeCy7, anti-CD19-PerCP, anti-ICAM-1-biotin, and streptavidin-APC (allophycocyanin), and analyzed on the LSRII flow cytometer.

### Supported planar lipid bilayers

GPI-linked or His-tagged forms of unlabeled I-E^k^ (200 molecules/ μm^2^) and Cy5-labeled ICAM-1 (300 molecules/ μm^2^), kindly provided by Michael Dustin (University of Oxford, UK), were incorporated into dioleoylphatidylcholine or Ni-NTA lipid bilayers as described [18, 19]. The bilayers were supported on a coverslip in a Bioptechs flow cell (http://www.bioptechs.com/Products/FCS2/fcs2.html) closed chamber system, and loaded by incubation with 100 μM MCC peptide in a PBS/citrate buffer at pH 4.5 and 37°C.

## Results

### Anti-IL-4 treated Th2 cells retain their multifocal synapse structure

Our prior work showed that Th1 and Th2 cells have very different immunological synapse structures [16]. To determine whether this difference was owing to culture in high concentrations of IL-4 during polarization of Th2 cells *in vitro* or truly owing to a difference between Th subsets, we used planar lipid bilayers containing fluorescent ICAM-1 and peptide-loaded MHC molecules to compare immunological synapses generated by Th2 cells that received either IL-4 or anti-IL-4 during secondary stimulation. The anti-IL-4 treated Th2 cells expressed preformed CD40L, while preformed CD40L was suppressed in Th2 cells not treated with anti-IL-4 (Fig 1A) [6, 20]. Lipid bilayer results compiled from three independent experiments showed that, while most of the Th1 cells displayed the expected bull’s eye structure [6], only 9% of IL-4-treated Th2 cells and 11% of the anti-IL-4 treated Th2 cells analyzed displayed the ICAM ring characteristic of the bull’s eye Th1 synapse, and most cells retained the characteristic multifocal synapse structure of Th2 cells (Fig 1B). This finding enabled us to compare the delivery of CD40L by the two helper T cell subsets with distinct immunological synapse structures.

### Th2 cells do not transfer CD40L to bystander B cells

**Fig 2:**
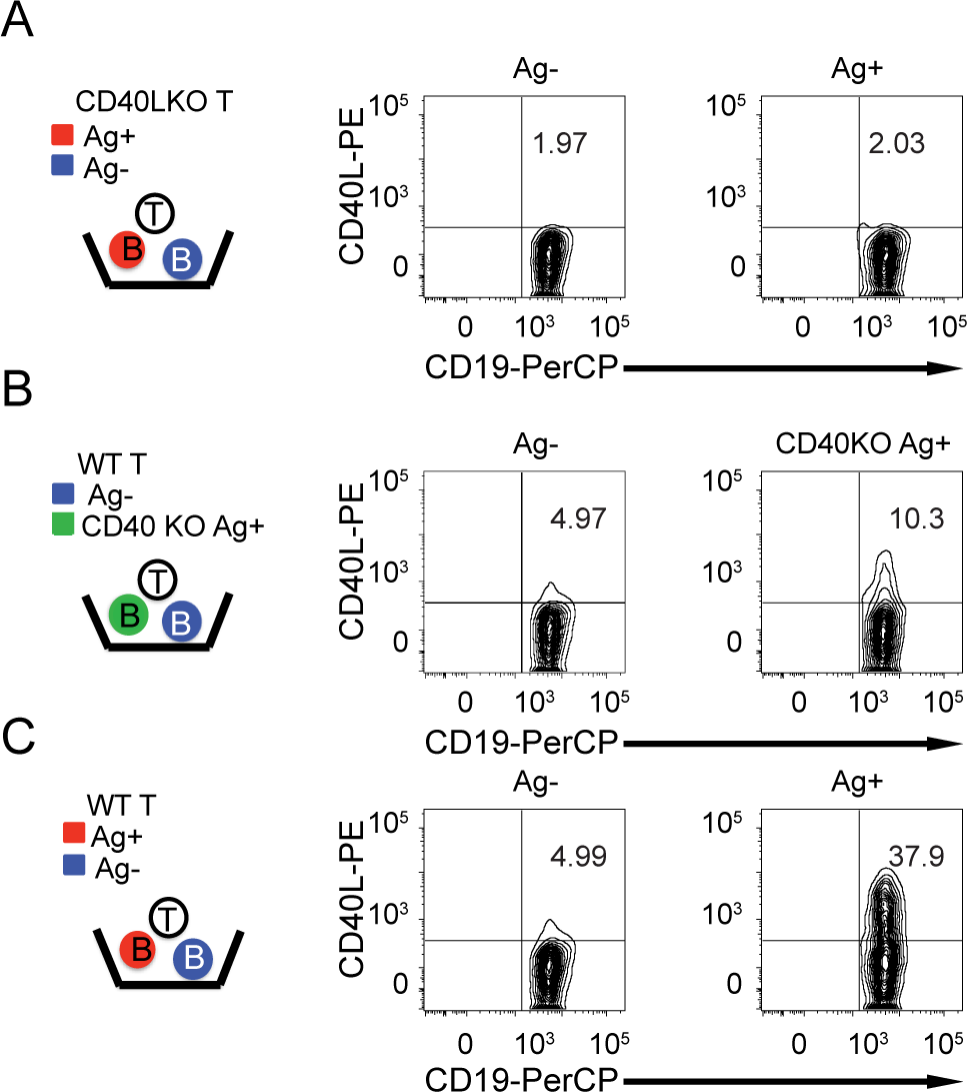
CD40L transfer by Th2 cells is antigen-specific and largely CD40 dependent. Antigen-pulsed B cells (Ag+, red) were mixed with unpulsed, bystander B cells (Ag-, blue) and cultured overnight with Th2 cells in the presence of 1 μg/ml fluorescent anti-CD40L. (**A**) CD40L knockout (CD40LKO) T cells do not transfer CD40L to antigen-pulsed B cells or bystanders. (**B**) CD40L transfer by Th2 cells to CD40 knock-out antigen-pulsed B cells (CD40KO Ag+) and CD40 sufficient bystander B cells. (**C**) CD40L transfer by Th2 cells to antigen-pulsed (Ag+) and bystander (Ag-) CD40 sufficient B cells. This experiment is representative of 3 independent experiments.

We have recently discovered that CD40L is transferred to antigen-presenting B cells following interactions with Th1 cells, and that transfer of CD40L correlates with B cell activation. We found that inclusion of fluorescently labeled anti-CD40L in the overnight cultures made detection of transferred CD40L much more apparent, and blocked transfer to and activation of bystander B cells that had not been antigen-pulsed [14]. If the ring structure of Th1 synapses were required for specific delivery of CD40L to antigen-bearing B cells, then Th2 cells, with multifocal synapses, would be less specific than Th1 cells in delivery of CD40L. We found that Th2 cells, like Th1 cells, transfer CD40L in an antigen-specific manner in the presence of fluorescent anti-CD40L, since antigen-pulsed B cells received CD40L and bystander B cells in the same cultures did not (Fig 2C). Using CD40L knockout T cells, we showed that CD40L comes from the T cells (Fig 2A), and using CD40 knockout B cells, we found that transfer to B cells is largely but not completely CD40 dependent (Fig 2B), as previously shown for Th1 cells [14]. Despite the multifocal synapse, Th2 cells do not transfer CD40L to bystander B cells over background levels under these conditions.

### Delivery of preformed CD40L by Th2 cells results in solely antigen-specific activation

**Fig 3:**
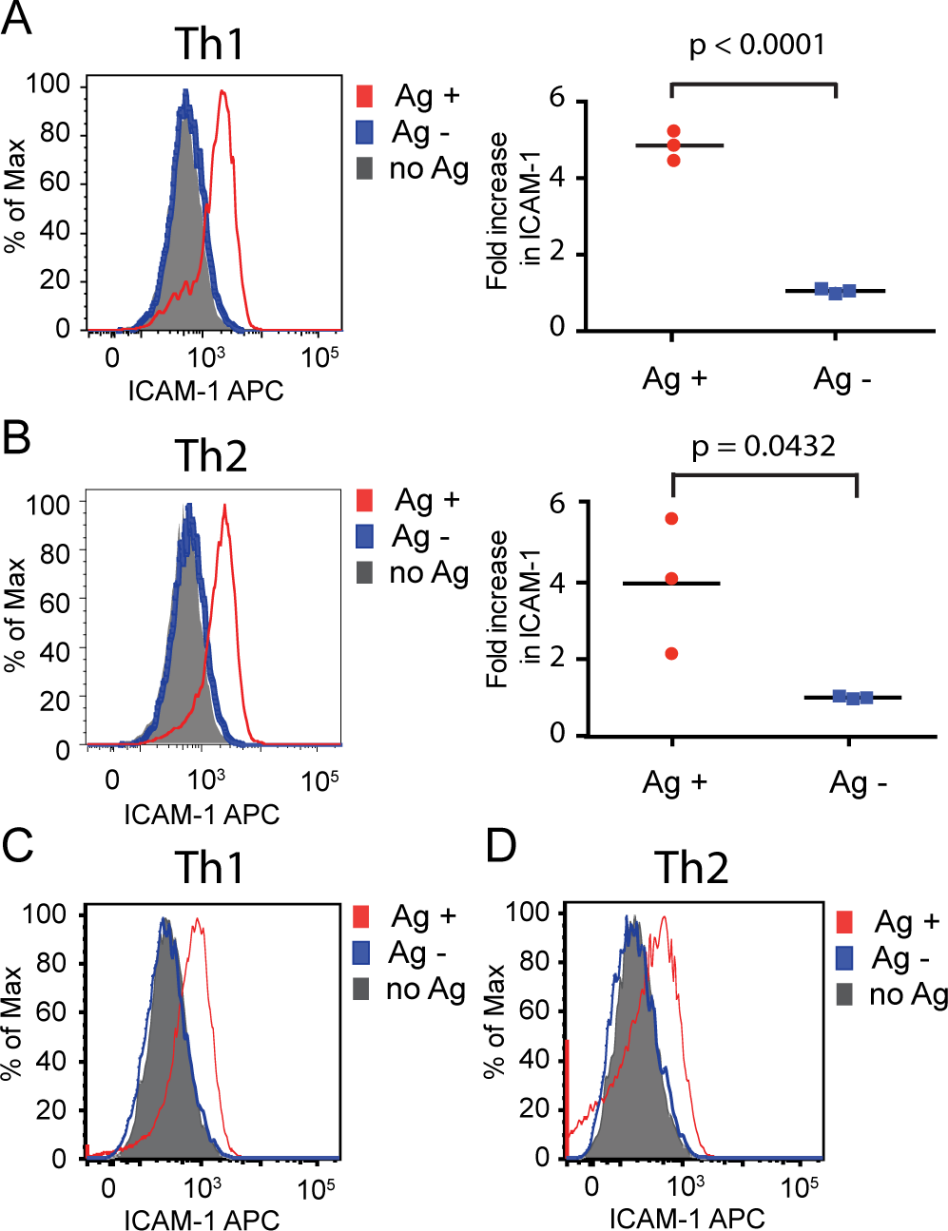
Th2 cells expressing preformed CD40L help only antigen-presenting B cells. Upregulation of ICAM-1 was measured on mixed antigen-pulsed (Ag+, red) and bystander (Ag-, blue) B cells following overnight incubation in a 12 well plate with Th1 (**A**) and Th2 (**B**) cells in the presence of CsA to block de novo CD40L synthesis. Representative histograms are shown in the left panels. ICAM-1 on B cells cultured with T cells but without antigen in the well (no Ag) is shown as solid gray histograms. The panels on the right show mean fold increase in ICAM-1 fluorescent intensity on antigen-pulsed and bystander cells compared to the control without antigen (set to 1) in three experiments. (**C, D**) The same experiment was performed with T cells, antigen-pulsed B cells, and bystander B cells spun together and cultured in a 96 well round-bottom plate to enhance cell contact. Th1 histograms are representative of three independent experiments. Th2 histograms are representative of two independent experiments. ICAM-1 levels for the control lacking antigen are shown in solid gray.

Previous studies showed that the small amount of preformed CD40L stored in helper T cells that can be delivered within minutes of TCR stimulation to the T cell surface is sufficient to activate antigen-presenting B cells [6, 11]. Therefore, we were particularly interested in B cell activation owing to preformed CD40L, and how this delivery might be affected by differences in immunological synapse structure. As in the previous studies, we used CsA to block *de novo* synthesis of CD40L, and measured ICAM-1 upregulation on the B cells as a sensitive and specific readout of CD40L-dependent T cell help for B cells [11]. We found that when CD40L delivery is limited to preformed CD40L by treating T cells with cyclosporine A, both Th1 and Th2 cells activated only antigen-presenting B cells and not bystander B cells (Fig 3A and 3B). When T cells and B cells were spun down together in a round-bottom 96 well plate overnight, increasing the likelihood of bystander activation, again both Th1 and Th2 cells activated only the antigen-pulsed B cells (Fig 3C and 3D). Therefore, the multifocal synapse structure of Th2 cells does not result in bystander activation when T cells are delivering limited amounts of preformed CD40L.

### Th2 cells preferentially activate antigen-presenting B cells when de novo CD40L synthesis is not inhibited

**Fig 4:**
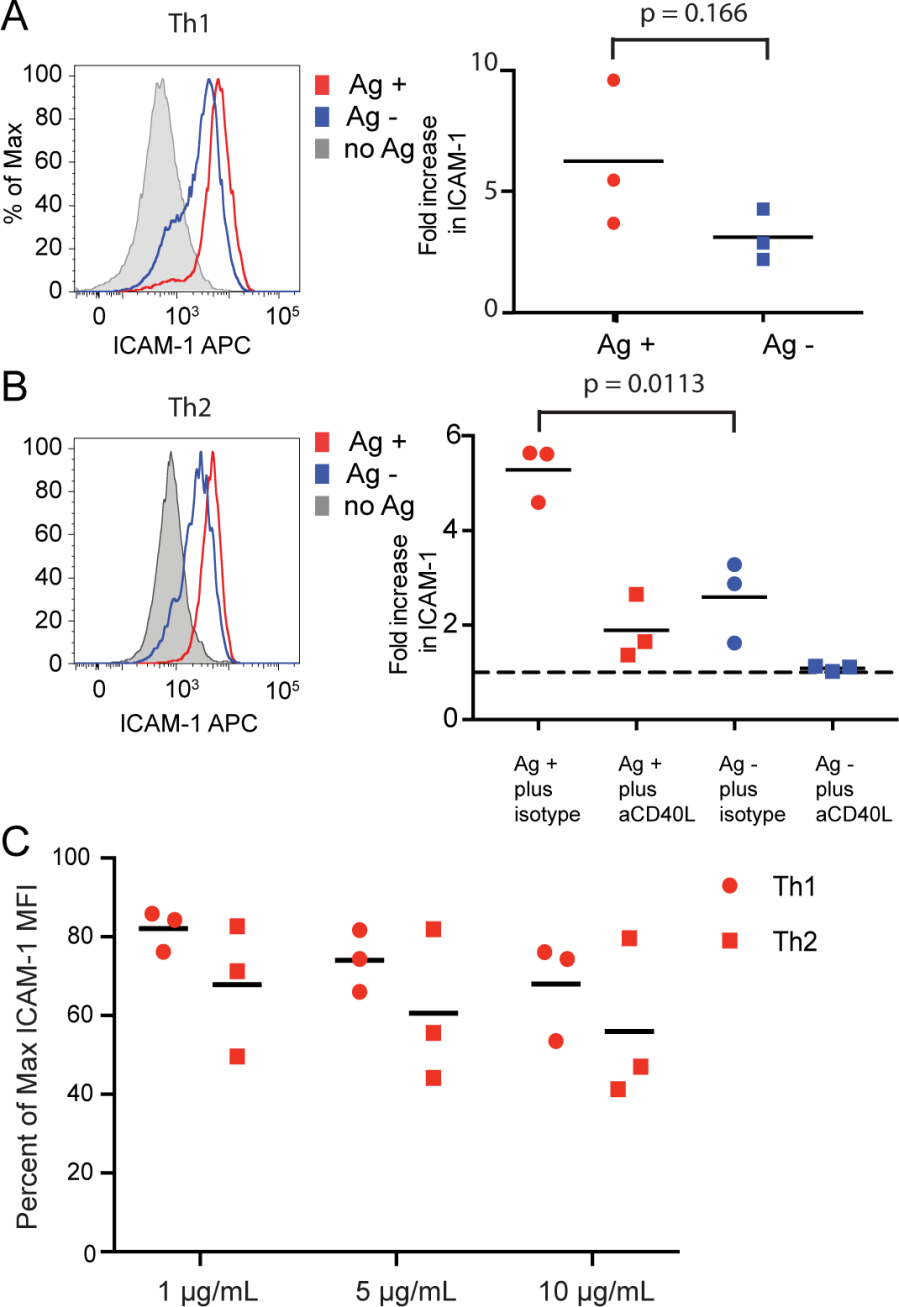
In the absence of anti-CD40L or CsA, Th2 deliver help preferentially to antigen-presenting B cells, and inhibition of Th2 help by anti-CD40L is comparable to that of Th1 cells. ICAM-1 was measured on antigen-pulsed (Ag+) and bystander (Ag-) B cells mixed and incubated overnight with Th1 (**A**) or Th2 cells (**B**). The inhibition of Th2 help by addition of anti-CD40L or isotype IgG control at 10 μg/ml is shown in (**B**). Results are shown for three independent experiments. (**C**) Inhibition of help for antigen-presenting B cells by Th1 and Th2 cells is shown for a range of concentrations of anti-CD40L. The graph shows the percentage of maximum ICAM-1 fluorescent intensity in the presence of anti-CD40L blocking antibody. Results of three independent experiments are shown.

Sustained interactions of Th cells with naïve B cells are required for B cell priming [21, 22] and allow sufficient time for *de novo* CD40L synthesis. Therefore, we tested bystander activation under conditions in which *de novo* CD40L production is not inhibited. *In vitro*, in the absence of anti-CD40L or CsA, it is well known that help is delivered to both antigen-presenting and bystander B cells, but our earlier studies with Th1 cells showed some preferential activation of the antigen-presenting B cells relative to bystanders [14]. Under these conditions, we found that Th2 cells, like Th1 cells, activate both antigen-presenting B cells and bystander B cells, but show preferential activation of antigen-presenting B cells (Fig 4A and 4B). Activation of bystander B cells by Th2 cells is CD40L-dependent, as show earlier for Th1 cells (11), since anti-CD40L completely inhibits bystander B cell activation (Fig 4B, right panel).

### Anti-CD40L can block activation as effectively in the Th1 synapse as the Th2 synapse

As mentioned above, low concentrations of a blocking anti-CD40L antibody completely prevent transfer of CD40L to bystander B cells (Fig 2) and activation of bystander B cells during overnight culture (14, Fig 4B). Higher concentrations of anti-CD40L incompletely block activation of antigen-presenting B cells by Th1 cells [14]. To test whether the Th2 multifocal synapse may be more accessible to blocking by anti-CD40L compared to the Th1 synapse with a bull’s eye ring of LFA-1 and ICAM adhesion molecules, we compared the ability of anti-CD40L to inhibit activation of antigen-presenting B cells cultured with Th1 or Th2 cells. Help by Th1 and Th2 cells was partially inhibited to a similar degree across a range of concentrations of anti-CD40L (Fig 4C), and the small difference in percent inhibition between Th1 and Th2 may be owing to somewhat higher level of CD40L production and help delivered by Th1 cells.

## Discussion

Although other functions of the immunological synapse have been proposed, the established function is to direct paracrine secretion of effector molecules to antigen-presenting target cells [23–26]. We show in this report that Th1 and Th2 cells are comparable in their ability to deliver T cell help in the form of CD40L to antigen-presenting B cells in an antigen-specific manner. Therefore, the multifocal, Th2 synapse is fully capable of antigen-specific T/B collaboration, and the well-organized, bull’s eye synapse of Th1 cells is not required for antigen-specific delivery of T cell help.

Pathways of secretion of effector molecules by T cells are complex. Antigen recognition results in polarization of the T cell and migration of the microtubule organizing center and the Golgi apparatus to a position just beneath the cell-cell interface with the APC [23, 25]. Cytolytic granules ride to the synapse on microtubules and are delivered to target cells through a well-characterized secretory zone in the center of the synapse [24]. Delivery of cytolytic granules to the immunological synapse is dependent on the extent of actin clearance in this interaction zone [27, 28]. Therefore, a bull’s eye synapse may be necessary for delivery of cytolytic granules, while small cytokines may be delivered as effectively within a multifocal synapse. Huse et al. [29] described two pathways of secretion of soluble cytokines involving different Rab and SNARE proteins. IFNγ from Th1 cells and IL-10 from Th2 cells were shown to be delivered in a focused manner towards the synapse, whereas TNFα and IL-4 were secreted multidirectionally. CD40L was not examined in that study. Recently, Choudhuri et al. visualized release of microvesicles containing TCR directly onto antigen-presenting supported bilayers through the immunological synapse [26]. Delivery of cytokines to the synapse in other kinds of extracellular vesicles has been described [30]. We showed that preformed, intracellular CD40L in effector CD4 T cells is colocalized with FasL, but not with LFA-1 [5], and that Rab27a, essential for killing by cytolytic T cells and NK cells, is not required for mobilization of preformed CD40L to the cell surface by antigen recognition [11].

T follicular helper (Tfh) cells are the most relevant T cell subset for analysis of T cell help for B cells [31–33]. Tfh cells interact with B cells in the specialized areas of lymph nodes and spleens called germinal centers where the processes of somatic hypermutation and isotype switching occur. It has been shown that tumor infiltrating Tfh cells may help to maintain CTLs at tumor sites, though they do not differentiate into cells that produce cytotoxic granules [34–36]. Therefore, if the function of the bull’s eye synapse is solely the delivery of cytolytic granules, Tfh, like Th2 cells, may have a multifocal synapse structure. Tfh cells were not analyzed in this study because effective conditions for inducing them *in vitro* have not been discovered [37]. Future studies will be necessary to determine the immunological synapse structure of Tfh cells and delivery of CD40L within this structure.
